# From a glimpse into the key aspects of calibration and correlation to their practical considerations in chemical analysis

**DOI:** 10.1007/s00604-023-06157-4

**Published:** 2024-01-09

**Authors:** Constantine Stalikas, Vasilios Sakkas

**Affiliations:** Department of Chemistry, University of Ioannina, 451 10 Ioannina, Greece

**Keywords:** Linearity assessment, Outliers, Error of predicted concentrations, Correlation and agreement, Standard addition, Practical example

## Abstract

In this tutorial review, we provide a guiding reference on the good practice in building calibration and correlation experiments, and we explain how the results should be evaluated and interpreted. The review centers on calibration experiments where the relationship between response and concentration is expected to be linear, although certain of the described principles of good practice can be applied to non-linear systems, as well. Furthermore, it gives prominence to the meaning and correct interpretation of some of the statistical terms commonly associated with calibration and regression. To reach a mutual understanding in this significant field, we present, through a practical example, a step-by-step procedure, which deals with typical challenges related to linearity and outlier assessment, calculation of the associated error of the predicted concentration, and limits of detection. The utilization of regression lines to compare analytical methods is also elaborated. The results of regression and correlation data are acquired by implementing the *Excel* spreadsheet of *Microsoft*, being perhaps one of the most widely used user-friendly software in education and research.

**Graphical Abstract:**

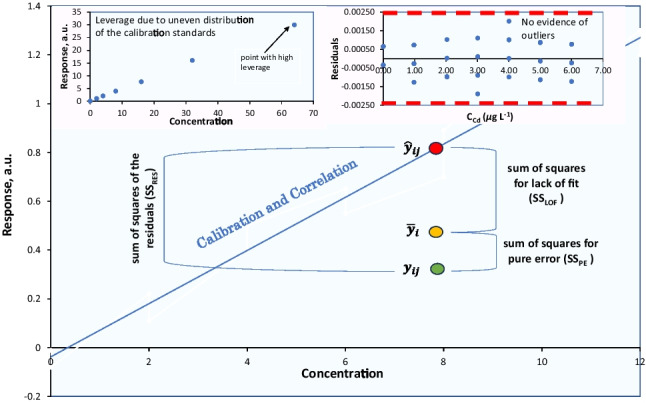

## Introduction

*Regression* and *correlation* are the most used techniques for investigating the relationship between two quantitative variables [1, 2]. There is, though, a key difference between them; regression is how one variable affects another and correlation measures the degree of a relationship between two independent variables (*x* and *y*). A good example of regression analysis is the construction of an analytical calibration curve, where the instrument response (the dependent variable) depends upon the concentration of the analyte (independent variable). In other words, if someone aims to analyze the effect of how an independent variable is numerically associated with a dependent variable, then the use of regression is mandatory [3]. The ultimate goal of calibration involves the prediction of the concentration of an analyte from a single instrumental response after setting up the above relationship between the values of known samples (i.e., standards with known amounts of analyte present) and instrument responses. *Calibration curves* (or *graphs or plots*) are the bread and butter of analytical chemistry, and their examination is an important step in any method validation or application.

Although the common name of the resulting plot is “calibration curve,” analytical chemistry researchers, typically, attempt fitting a linear function. Calibration, typically, involves the proper preparation of a set of standards containing a known amount of the analyte of interest, measurement of the instrument response for each standard, and establishment of the relationship between them. Based on a certain number of measurements of standards, two fitting techniques for the linear regression model can be established to set up the calibration curve and estimate the slope and the intercept of a linear calibration model: the ordinary least-squares (OLS) and weighted least-squares (WLS) [4, 5]. The testing of the reported linearity of a calibration curve should be an everyday task in routine analytical operations. While a great deal of effort is put in the selection and calibration of an analytical instrument, the choices behind curve calculation are usually overlooked. However, neglecting the consideration of statistics of calibration can lead to unfortunate conclusions as there is no advanced instrumentation or additional measurements that can rescue sound data from an erroneously built calibration curve. A calibration curve should either confirm the analyte–response relationship or raise an alert of the presence of a problem, which should properly be addressed.

Critical to the success of calibration and correlation is the understanding of the limitations and statistics used to set up a curve. The aim of this tutorial review is to provide a good practice guide in building calibration and correlation experiments and to explain how the results should be evaluated and interpreted. It centers on calibration experiments where the relationship between response and concentration is expected to be linear, although many of the principles of good practice described can be applied to non-linear systems, as well. First, we provide a general-case argument for the minimum number of standards required by regulatory guidance. Then, we examine the poor success rate of simple outlier detection in calibration curves using equidistant (linear) and logarithmic scale standard spacing, as well as we look into the significant risks associated with extrapolating the curve (where appropriate) beyond the linear response region. To enhance the understanding of this significant field, we present, through a practical example, a step-by-step procedure dealing with typical challenges related to regression, outlier assessment, procedures for linearity testing, calculation of the associated error of the predicted concentration and the limits of detection. The utilization of the concepts of correlation and agreement to compare analytical methods is also elaborated. The regression data results are acquired by utilizing the *Excel* spreadsheet of *Microsoft*, being perhaps one of the most widely used user-friendly software in educational settings.

## Handling calibration data sets

One of the first questions analysts often ask is: “How many calibration standards do we need to measure and what is the number of replicates at each calibration level?” Before answering this question, the purpose of the calibration experiment must be defined. It is necessary to make a distinction between the calibration of a measurement system and the check of the validity of the calibration of a measurement system. To minimize the risk of error associated with improper calibration of a measurement system, international guidance dictates a minimum number of calibrators and the threshold at which a measurement becomes an outlier. Regulatory guidance provides the minimum required number of standards to establish the calibration curve. For an assessment of the calibration function, as part of a method validation, for example, standards with at least seven different concentrations should be included. The EURACHEM Guide “The Fitness for Purpose of Analytical Methods” and the draft guidance from the USFDA mandate a minimum of seven calibration standards—six plus zero concentration standards—to perform the calibration [6, 7]. Other documents lay down a different number of calibration levels. For example, ISO standard 15,302:2007 specifies four calibration levels and Commission Decision 2002/657/EC stipulates at least five concentration levels (including blank) for the construction of a calibration curve [8], as a minimum requirement for an assessment of the calibration function. ISO standard 8466–1:1990 demands ten calibration levels [9]. However, these requirements do not explain why the curve should be drawn with this number of points and not with more or less than that. The sample with zero analyte concentration should definitely be included as it allows us to gain better insight into the region of low analyte concentrations and detection capabilities.

The design of calibration experiments and the number of calibration levels depend very much not only on the purpose of the experiment but also on the existing knowledge. Less knowledge about the shape of the calibration functions requires performing initial assessment measurements on more concentration levels. Ideally, the calibration range should be drawn so that the concentrations of the analyte in the test samples fall in the center of the range, where the uncertainty associated with the predicted concentrations is minimized. It is also useful to make at least triplicate independent measurements at each concentration level, particularly at the method validation stage, as it allows the precision of the calibration process to be evaluated, at each concentration level. Analyte calibration solutions should be prepared from a pure substance with a known purity value or a solution of a substance with a known concentration.

**Fig. 1:**
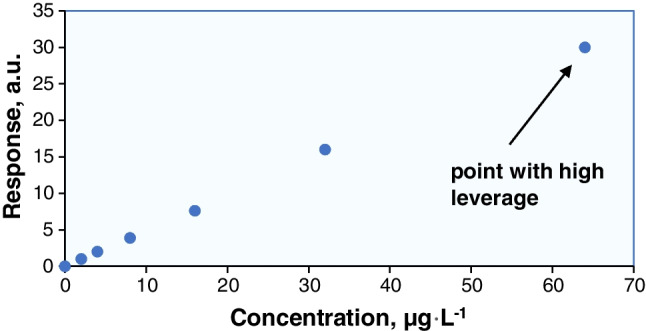
Leverage due to uneven distribution of the calibration standards

The standard concentrations should not only cover the range of concentrations encountered during the analysis of test samples but also, they should be evenly spaced across the range (for wide calibration ranges, partial arithmetic series could be considered). However, the risk of *leverage* arises from the introduction of error into the measurement in the calculated curve, even in the absence of an outlier (vide infra). Leverage can be a concern if one or two of the calibration points are far from the others along the *x*-axis (near the ends of a calibration curve), where any error has a disproportionate effect on the curve. Even if these points are not outliers, they may have a leverage to a certain degree. In other words, a relatively small error in the measured response will have a significant effect on the position of the regression line. Often, this situation arises when calibration standards are prepared by sequential dilution of solutions. The procedure frequently employed in the preparation of calibration standards is to prepare the most concentrated standard and then dilute it by, say, 50%, to obtain the next standard. This standard is subsequently diluted by 50% and so on (e.g., 64 μg L^−1^, 32 μg L^−1^, 16 μg L^−1^, 8 μg L^−1^, 4 μg L^−1^, 2 μg L^−1^). This procedure is not recommended as, in addition to the lack of independence, the standard concentrations will not be evenly spaced across the concentration range, leading to a leverage (e.g., the concentration of 64 μg L^−1^ in Fig. 1). As a result, the calculated slope and intercept might be disproportionally affected by that data point.

## Linearity in calibration and its misinterpretation

Linearity is an important feature for any analytical method. If the calibration function is linear, then, the estimation of the equation is easier and errors in estimating the concentrations of unknown samples from the calibration equation are likely to be smaller. The *correlation coefficient (r)* of a calibration graph or the *R-squared (R*^*2*^*)* or *coefficient of determination* is usually employed as an indicator of linearity, by inspecting its closeness to 1. Strictly speaking, *r* is a measure of the relationship between two variables *x* and *y*. Its use in calibration, though, is based on a widespread misunderstanding; if the calibration points are clustered around a straight line (and this is not the unique case), the experimental value of *r* will be close to unity. However, the opposite is not true. The International Union of Pure and Applied Chemistry (IUPAC) discourages the use of *r* to assume linearity in the relationship between concentration and analytical response. This is expressed by the excerpt from Ref. [4]: “... the correlation coefficient, which is a measure of the relationship of two random variables, has no meaning in calibration..*.*” Furthermore, when a new analytical method is developed, the guide for authors of the *Journal of Chromatography* Α explicitly states that “claims of linearity should be supported by regression data that include slope, intercept, standard deviations of the slope and intercept, standard error and the number of data points; correlation coefficients are optional.” That is, the criterion of *r* to provide a measure of the degree of linear association between concentration and signal is weak.

In view of the above, the perception of linearity based on the criterion of correlation coefficient has been overturned by relevant statistical tests, in the last years. The assessment of the linearity can be carried out by resorting to the analysis of variance (ANOVA) of the calibration data [10]. In this methodology, a comparison of the so-called lack-of-fit (LOF) variance with the squared pure error is made through an *F*-test (See the “Practical example” and “Procedures for linearity assessment” section); however, it is essential to consider the error components of the regression. The prediction error for each data point can be measured as the difference between the observed response (${y}_{ij}$) and the predicted response (${\widehat{y}}_{ij}$), i.e., ${y}_{ij}-{\widehat{y}}_{ij}$. To assess the overall prediction error, we calculate this difference for every data point, square it, and then sum all these squared differences, resulting in a term known as the “sum of squares of the residuals (SS_RES_).” The degrees of freedom (*d.f.*) associated with SS_RES_ is [*p (concentration levels)* × *n (replicates)*] − 2 since we estimate the slope and the intercept (i.e., two parameters).

**Fig. 2:**
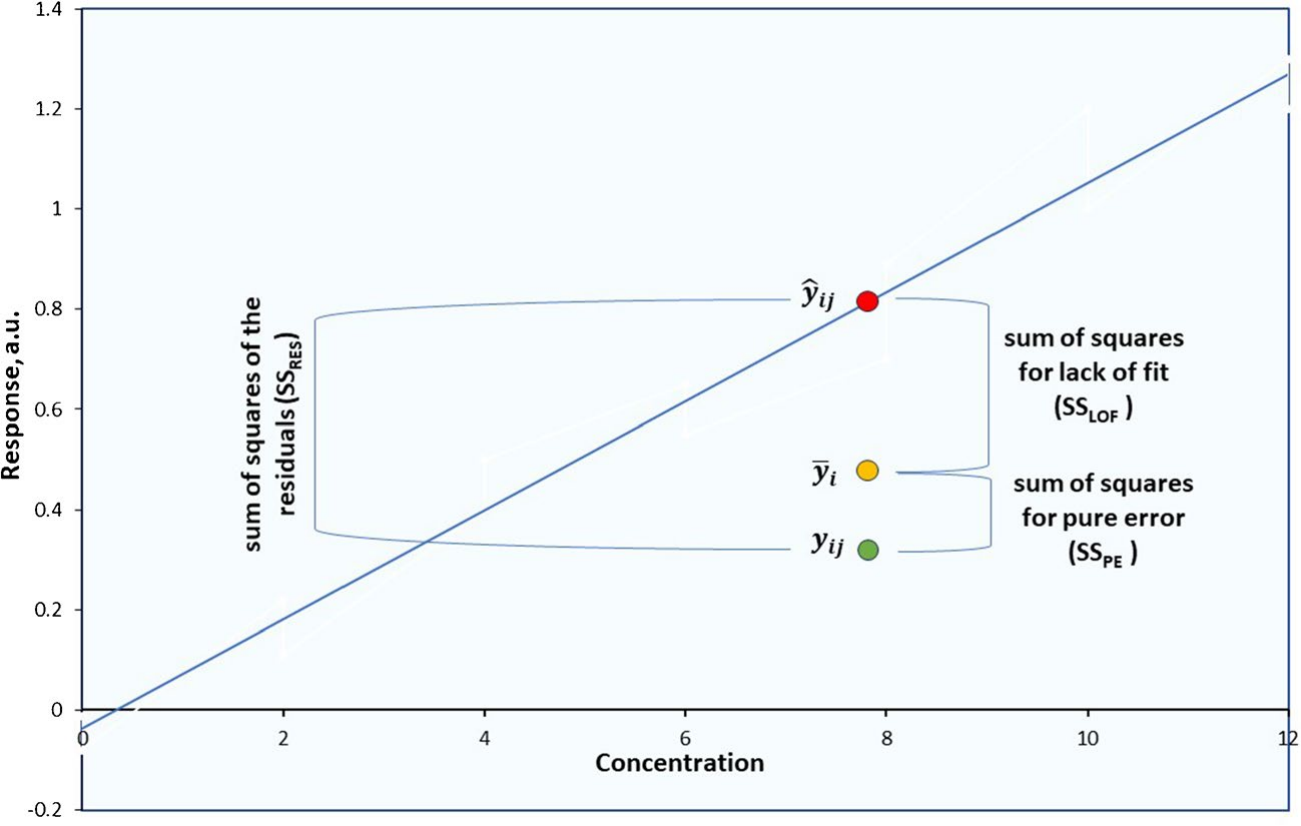
Error components in regression analysis

This residual error, SS_RES_, can be decomposed into two constituent components: the “sum of squares for lack of fit (SS_LOF_)” and the “sum of squares for pure error SS_PE_,” e.g., SS_RES_ = SS_LOF_ + SS_PE_ (Fig. 2).

When a line effectively fits the data, it implies that the average of observed responses ${\overline{y} }_{i}$ at each *x*-value closely aligns with the predicted response *(*${\widehat{y}}_{ij}$*)*, for that specific *x*-value. Consequently, to assess the extent to which the overall error arises from model inadequacy, we gauge the deviation between the average observed response at each *x*-value and the predicted response for each data point, i.e., ${\overline{y} }_{i}$ − ${\widehat{y}}_{ij}$ (Fig. 2). To measure the complete lack of fit of the model, we calculate this distance for every calibration point, square it, and sum all these squared differences to obtain the SS_LOF_ (associated with *p*-2 degrees of freedom).

To assess the portion of the overall error attributed solely to random fluctuations, we examine the extent to which each observed response (${y}_{ij}$) deviates from the average observed response (${\overline{y} }_{i}$) at each corresponding concentration (*x*-value), i.e., ${y}_{ij}-{\overline{y} }_{i}$. Similarly, the total pure error is calculated by summing the squared differences for each calibration point to get the SS_PE_ (*p* × *n* − *p* *d.f.*). When the respective sum of squares is divided by their associated degrees of freedom, the mean squares (MS) are obtained. It is those mean squares from which we can calculate the *F*-statistic, as follows:

$$F = \frac{MS\left(LOF\right)}{MS \left(PURE\; ERROR\right)}$$

It is advisable that readers, for further reading, peruse the excellent brief reports edited by the Analytical Methods Committee of the Royal Society of Chemistry [11] and the textbook “Calibration and Validation of Analytical Methods” edited by Mark T. Stauffer [12], which seek to introduce the readers to current methodologies of analytical calibration.

## Ηomogeneity and non-homogeneity of variances

Most of the reports in the literature refer to OLS, which practically, should be only used when experimental data have constant variance (homoscedasticity). In contrast, WLS is more appropriate when the variance varies (heteroscedasticity) or in other words, when every calibration point does not have an equal impact on the regression. There are several tests for homogeneity of more than two variances [13]. A simple way for testing homoscedasticity is to plot the residuals calculated from the straight line obtained by using the OLS method. A horizontal band of residuals indicates constant variance and unweighted least squares regression is recommended. A trumpet-shaped opening toward larger values signifies increasing variability as concentration increases.

From a practical viewpoint, when a narrow concentration range is considered, the unweighted linear model is usually adapted while a larger range may require a weighted model. If the weight is estimated incorrectly, the calculated estimators of regression coefficients (slope and intercept), being sensitive to extreme data points will be biased, with a concomitant negative impact on the predicted concentration intervals for real samples. It is noted that ignoring the inhomogeneity of variances will not sacrifice much statistical reliability when working in the mid-range of the calibration curve. Nonetheless, the WLS can reduce the limit of quantification and enable a broader linear calibration range with higher accuracy and precision, especially for bioanalytical methods. Depending on the characteristics of the data set, the weighting factors can be employed in a number of different ways [14]. The incorporation of heteroscedasticity into the calibration procedure is recognized by ISO and USFDA; the latter recommends that “the simplest model that adequately describes the concentration–response relationship should be used. Selection of weighting and use of a complex regression equation should be justified” [15]. Raposo, in his tutorial review, provides an illustrative example selected from the literature that best suits the weighting approach in calibration [5]. A more detailed examination of this topic is beyond the scope of this review.

## Non-linearity

**Fig. 3:**
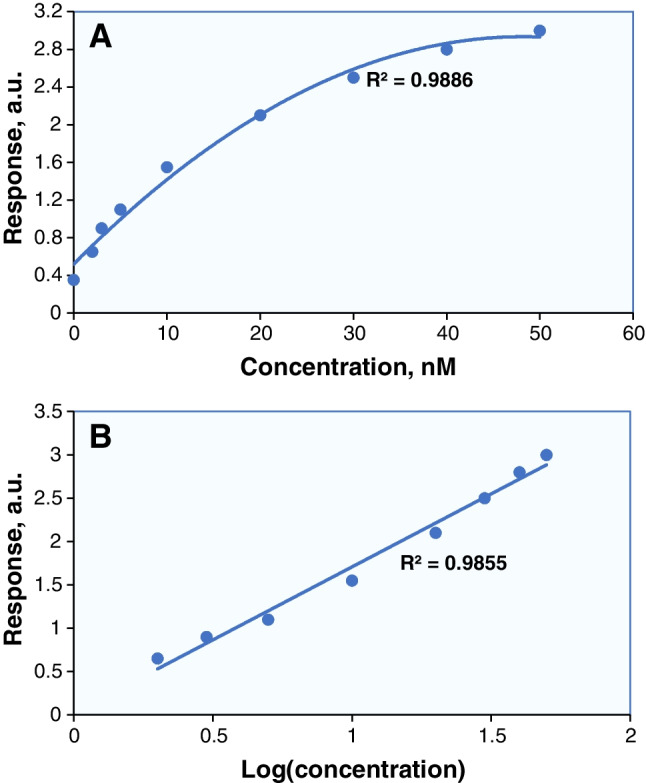
Calibration data set in standard plots with linear axes showing response plotted against **A** concentration and **B** logarithm of concentration. Graph **B** is often incorrectly used to prove linear dependence between analytical response and concentration

It may be the case that several analytical methods exhibit good response over a broad concentration range (i.e., several orders of magnitude). Because of this behavior, it is helpful to construct a double logarithmic plot based on the raw data. Importantly, the resulting plot serves the purpose of proving the method response over this broad concentration range but not of fitting linear function to the data sets. In other cases, non-linear dependence of analytical response on concentration is likely to appear, for instance, in analytical methods based on electrochemistry (Fig. 3). The method that leads to such a calibration data set may have unacceptably low or very low sensitivity (note the low slope in the concentration range above 10 nM in the raw data calibration plot of Fig. 3A). To cope with this situation, some researchers choose to tap into the logarithms of concentration values and the analytical response (Fig. 3B), thus claiming linearity of the method over the extended concentration range. Using raw data or data after logarithmic transformation primarily depends on analytical principles.

Arguably, associating the analytical response with the logarithm of concentration in handling calibration data sets may not be the best choice, as it can easily lead to misinterpretation of the analytical performance. A legitimate practice in this case is to consider displaying calibration data sets in plots with linear axes (equidistant scale) fitted with logarithmic or other nonlinear functions. Nonetheless, when the non-linear relationship is fitted for a small number of concentration levels this makes the process unreliable. Any curve that includes a non-linear response should have sufficient additional points between the upper limit of linearity and the upper limit of quantitation to describe the inflection point. Unless a great number of concentration levels are included in the data set, the plateau interval within a fitted nonlinear function cannot be used for quantitative analysis.

In 2020, P.L. Urban published a Perspective on the dependence between analytical response and logarithm of concentration, with the deterring title “Please Avoid Plotting Analytical Response against Logarithm of Concentration” [16]. The author puts forward a well-argued case for proper data treatment, where a non-logarithm of concentration is displayed in the *x*-axis of the calibration plot, threatening bad results if someone does not follow it. Others, in defense of the application of logarithmically transformed data, claim that enzyme-catalyzed reactions or electrochemical data in logarithmic form are more appropriate for function fitting [17].

## Outliers’ assessment in linear regression

**Fig. 4:**
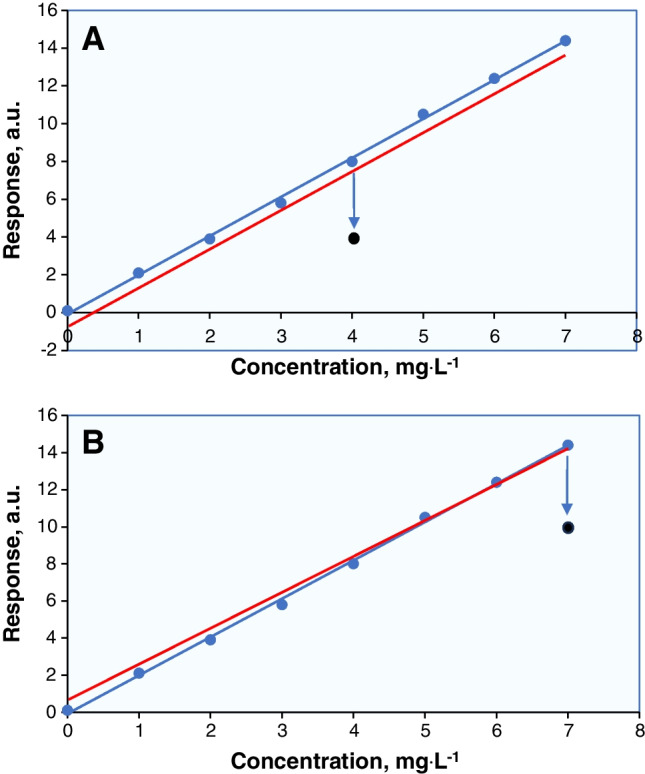
**A** The outlier laid in the middle of the calibration range introduces bias (red line). **B** Leverage caused by an outlier laid in the uppermost part of the calibration curve

An outlier is an experimental measurement that is significantly different from the rest of the entire data set. In the case of calibration, an outlier appears as a point which is well apart from the trend of the other calibration points and introduces a *leverage* or *bias* into the position of the line. Once in the middle of the calibration range, the outlier can shift the regression line up or down (Fig. 4A). The slope of the line will approximately be correct but the intercept will be wrong. In this case, a bias is introduced. An outlier at the extremes of the calibration range will change the position of the calibration curve by tilting it upwards or downwards (Fig. 4B). The outlier is said to have a degree of leverage.

After estimating the regression parameters, the plot needs to be examined to identify any data points that deviate significantly from the remaining data set (considering the assumption of linear calibration). For this purpose, initially, the residuals (*y*_*i*_ − $\widehat{y}$) are calculated and graphed against their corresponding concentration levels. Two horizontal dotted-dashed lines, representing ± *t* (0.05, p–2) ×* S*_*y*/*x*_, are utilized to denote the permissible deviation for each individual point in the residual plot. Any calibration data point(s) that lie outside these lines are considered qualitative outliers. Details are given in the “Practical example” and “Assessment of outliers” section.

With respect to identifying outliers in the case of fitting curves with nonlinear regression, a method has been proposed, which combines robust regression and outlier removal [18]. Although this is not an easy task, the analysis of simulated data demonstrated that this procedure identified outliers from nonlinear curve fits with reasonable power and few false positives.

## Standard addition(s) method

The use of pure standard solutions to establish the ordinary calibration graph assumes that there is no reduction or enhancement of signal by other matrix components of real samples. Hence, the so-called *direct calibration method* of analysis can be applied. However, in many areas of analytical chemistry, this assumption is not valid as matrix effects can occur even with methods that have the reputation of being relatively free from interferences. A solution to this problem is that all the analytical measurements, including the construction of the calibration graph, can be performed using the sample itself, thus applying the *standard addition(s) method*. Matrix effects should be ascertained previously; this can be done by testing the statistical equality of the slopes of the lines arising from the direct calibration and standard addition methods. If slopes are demonstrated to be statistically different at a certain confidence level (and the adequate number of degrees of freedom), the use of the standard addition method is undoubtedly the most favorable choice [19].

Note that sound evidence of linearity of the direct calibration method is a requisite for the use of the method of standard addition and the extrapolation involved. Also, standard addition cannot be applied if the intercept of the regression equation estimated by pure standard solutions in the direct calibration method is not zero, or close to zero (the zero-intercept assumption is seldom plausible). Unless the intercept is zero, the method will show a positive error compared with the true concentration of the target in the sample.

Based on the above, once it has been established that the linearity of the direct calibration method fits the data, a statistical test should be carried out for zero intercept (see the “Practical example” and “Procedures for linearity assessment” section). This can be judged by inspecting the confidence interval of the intercept *a*, i.e., *a* ± *t × s*_*a*_ (*s*_*a*_ is the standard error of *a* and *t* the two-tailed Student’s with *n*–2 degrees of freedom). If zero value is included within the confidence interval ± *t × s*_*a*_, then, the intercept is statistically zero. Provided that zero-intercept has been demonstrated, the standard addition approach adheres to the following procedure: (i) take several portions of the (treated) solution (six or seven at a minimum) and add a known amount of the analyte to each of them; (ii) the amounts added are (almost) evenly spaced from zero to maximum. Importantly, the total volume of all the treated solutions should be kept constant; (iii) the responses are measured, and the original concentration is estimated by extrapolation of the line to zero response.

Despite its capacity, the implementation of standard addition implies certain limitations. The extrapolation causes the technique to perform poorly in narrow (linear) calibration ranges. When carrying out such an experiment it is, therefore, recommended to add several times the original analyte concentration. Also, extrapolation degrades the precision compared with direct calibration where interpolation is exploited and hence, the uncertainty is generally increased. More details about its application are beyond the scope of this review.

## Standard deviation *vs* standard error

From a properly constructed calibration plot, the analyst expects that a reliable calculation of the concentration of analyte in tested samples can be made. No quantitative result is of any value unless it is accompanied by a realistic estimate of uncertainty, i.e., the range within which the true value of the quantity being measured should lie. At this stage, the *residual standard deviation* (or *error*) can be used as an estimate of the uncertainty in predicted concentration values. This is due to the precision of measurements (as represented by the residual standard deviation) being an important factor in assessing the uncertainty. Additionally, the regression model can be employed for estimating the limit of detection of the analytical procedure (see the “Practical example” and “Calculation of a concentration, its error, and the limits of detection” section). Hence, the random errors in the slope (*S*_*b*_) and intercept (*S*_*a*_) values hold significance (see the “Practical example” and “Assessing the scatter plot and performing regression analysis” section). “Standard error of mean” (SEM) and “standard deviation” (SD) are employed in different contexts and have different interpretations and calculations; however, they are often confused and misused and for this reason, they deserve discussion [20, 21]. The SD may be a good estimate of the variability of the population from which the data (i.e., the statistical sample) was drawn. For normally distributed data, about 99% of them will have values which lie within 3 × SD of the mean value while the other 1% is scattered above and below these limits. Evidently, in case that widely scattered measurements are to be expressed, the standard deviation should be quoted.

As the sample mean varies from statistical sample to sample in a population, the way this variation occurs is expressed by the “sampling distribution of the mean.” Here, the SEM is a type of standard deviation, which expresses the precision of the sample mean and is calculated by the simple relation:

$${\text{SEM}}={\text{SD}}/\sqrt{\mathrm{sample\; size}}$$

The SEM is always smaller than the SD; as the sample size increases the standard error decreases. From a practical viewpoint, if the aim is to obtain an insight into the uncertainty around the estimate of the mean value of the measurements—this is almost invariably the case in analytical chemistry—reporting the SEM is the most useful and reliable way of calculating a confidence interval. For a large sample, a 95% confidence interval is obtained as values of 1.96 × SEM either side of the mean. To report a 95% confidence interval instead of a 99% one is only a matter of choice, and has become a convention, related to calling statistically significant a *p*-value lower than 0.05. the analyst/researcher should appreciate that the contrast between these two terms reflects the distinction between description statistics (i.e., SD) and inference statistics (i.e., SEM). Standard deviation is the degree to which individual values within a statistical sample differ from the sample mean. In contrast, SEM gauges how close the sample mean is likely to be to the population mean.

Finally, in many publications/analytical reports, the sign ± is used to join the SD or SEM to an observed mean—for example, 13.4 ± 2.3 or 13 ± 2. However, this notation does not give an indication of whether the second figure is the SD or SEM. Analysts/researchers are advised to indicate clearly whether standard deviation or standard error is being quoted.

## Correlation *vs* agreement

As mentioned above, correlation allows researchers to know the association or the absence of a relationship between two variables. When these variables are correlated, we can measure the strength of their association. Tasks pertinent to the correlation in analytical chemistry often involve demonstrating a degree of association between analytical methods. These may be carried out by investigating a relationship between a new method and an official/reference/alternative interference-free method. When researchers seek to report this association, correlation or agreement is often used. However, the term “correlation” as a synonym of “agreement” can be misleading in any field of research [22]. This part aims to clarify the definition of these two terms in method development.

Correlation coefficient *r* does not provide any information on the agreement between two variables. Under certain conditions, the magnitude of *r* only provides information on how close the points lie to the regression line (see above, the “Linearity in calibration and its misinterpretation” section). The correlation analysis assumes that the distribution of one variable does not depend on the other, which is the case when comparing two different analytical methods. Also, *r* does not assume normality but it does assume homoscedasticity, i.e., constant variance. Inspection of the available data can reveal whether the correlation is linear or non-linear. In this context, a scatterplot of data should always be the first step before interpreting a correlation coefficient, to avoid incorrect assumptions about the relationship between the variables of interest.

When calculating *r*, statistical software reports a *p*-value which represents the chance that a significant linear correlation does not exist between the variables (this is the null hypothesis). A significantly non-zero *r* means that there is a dependence between *x* and *y*. Here, “significant” means how far we would expect *r* to be from zero and depends on both the number of measurements, *n*, and the distribution of each. A low *p*-value (≤ 0.05) provides evidence that the measured *r* represents a significant correlation between two variables. When a correlation exists, linear regression enables the calculation of the equation that minimizes the distance between the fitted line and all the data points in the sample. The *R*-squared (*R*^2^) or coefficient of determination commonly used in analytical chemistry, is another measure often encountered in linear regression analysis.

The term agreement is distinct; it is used to assess whether the measurements by two analysts or two different methods yield similar results. In the case of comparing the concentration of an analyte via two methods, we are measuring the same analyte in the same sample with different methods. Τhe two values may be correlated but do not necessarily agree. In this case, *r* is not sufficient. Even if the two methods are likely to be highly correlated with an *r* approaching unity, this does not provide concluding evidence that the methods agree. A high *r* value may indicate agreement but this remains false without further analysis to assess potential biased results. Again, by inspecting the relationship on a scatter plot it may be easy to see if they demonstrate poor agreement with significant bias.

Ordinary least-square regression could potentially be used to assess agreement; however, this regression assumes that one method is error-free and this is not true in most settings. When agreement between paired measurements is required, a *Bland–Altman plot*, also referred to as a *difference plot*, is a straightforward and reliable alternative to a scatter plot [23]. Practically, it is about a plot of the difference between two measurements on the *y*-axis (Method 1 minus Method 2) against the mean of the two measurements on the *x*-axis ({Method 1 + Method 2}/2). This simple plot reveals any bias between measurements, which is the difference between Method 1 and Method 2. Limits of agreement (LOA) are plotted as separate lines, demonstrating the range within which 95% of the differences between one method and the other are included. These limits are expressed as: mean observed difference (*m*_*d*_) ± 1.96 × standard deviation of the observed differences (*sd*_*d*_). Broad LOA or values that consistently fall outside these bounds is an indication of a lack of agreement between the two methods (see the “Practical example” and “Comparison of analytical methods” section).

As alluded to above, correlation is not synonymous with agreement. Correlation refers to the presence of a relationship between two different variables, whereas agreement refers to the concordance between two measurements of one variable. Two sets of measurements, which are highly correlated, may have poor agreement. However, if the two sets agree, they will surely be highly correlated.

## Practical example

To showcase the suitability of the suggested approach, we utilize data from a method that has been internally validated for the determination of Cd in natural water (Table 1).

**Table 1:** Calibration data for Cd determination in natural water

No	Cd Conc – *μ*g⋅L^−1^ (Concentration levels: *p* = 7)	Absorbance (Replicates: *n* = 5)
1	0.000	− 0.001
2	0.000	− 0.002
3	0.000	− 0.001
4	0.000	− 0.001
5	0.000	− 0.002
6	0.980	0.010
7	0.980	0.010
8	0.980	0.009
9	0.980	0.009
10	0.980	0.008
11	2.030	0.020
12	2.030	0.022
13	2.030	0.020
14	2.030	0.020
15	2.030	0.021
16	3.010	0.030
17	3.010	0.032
18	3.010	0.033
19	3.010	0.032
20	3.010	0.031
21	4.005	0.043
22	4.005	0.042
23	4.005	0.044
24	4.005	0.045
25	4.005	0.044
26	5.005	0.053
27	5.005	0.053
28	5.005	0.055
29	5.005	0.055
30	5.005	0.054
31	6.000	0.065
32	6.000	0.066
33	6.000	0.066
34	6.000	0.065
35	6.000	0.064

### Assessing the scatter plot and performing regression analysis

**Fig. 5:**
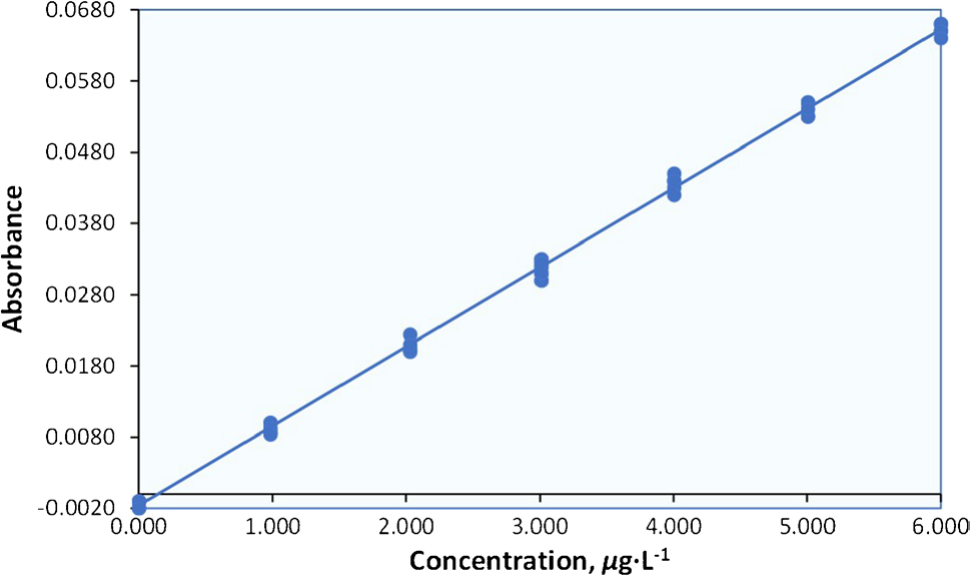
Calibration plot of response (Absorbance) versus Cd concentration (*μ*g⋅L^−1^)

The process that allows for a quick identification of any issues with the calibration data is the visual inspection of the calibration plot (Fig. 5).

Based on the findings, a typical approach would be to assume that the response is linearly correlated to the concentration. Regression determines the optimal values of slope (indicated as “*b*”) and intercept (indicated as “*α*”), that best describe the linear relationship between the analyte level (*x*) and the analytical signal (*y*). Typically, regression analysis is conducted using specialized software provided with instruments or popular packages like *Excel* (Table 2).

**Table 2:** Output of *Excel* regression

Regression statistics						
Multiple *R*		0.99920187				
*R* square		0.99840437				
Adjusted *R* square		0.99835602				
Standard error		0.00091869 ^*^(*S*_***y/x***_**)**				
Observations		35				
ANOVA						
	*df*		*SS*	*MS*	*F*	Significance *F*
Regression	1		0.01742712	0.01742712	20,648.52	9.72946E-48
Residual	33		2.78516E-05	8.43989E-07		
Total	34		0.017454971			
	Coefficients			Standard error	*t* stat	*p*-value
Intercept	− 0.00165967			0.000280046 ^*^(*S*_***a***_**)**	− 5.926421333	1.2E-06
*X* variable 1	0.01114682			7.75723E-05 ^*^(*S*_***b***_**)**	143.6959391	9.73E-48
	Lower 95%			Upper 95%		
Intercept	− 0.002229431			− 0.001089914		
*X* variable 1	0.010989002			0.011304646		

According to the *Excel* spreadsheet output, the instrumental response is linearly related to the Cd concentration (independent variable-*x*) based on OLS regression, in the form of *y* = *b* (± *S*_*b*_) *x* + *α* (± *S*_*a*_), as follows (with rounding to an appropriate number of significant digits):

1 $$y = 0.01115\; (\pm\; 0.00008)\; x - 0.0017\; (\pm\; 0.0003)$$

### Assessment of outliers

The differences between the observed values (*y*_*i*_) and the predicted values ($\widehat{y})$ (Table 3) are computed or can be generated from spreadsheet software programs.

**Table 3:** Responses and residuals from data of Table 1 after OLS regression

No	Response (*y*_i_)	^*a*^Predicted response ($\widehat{y}$)	Residuals (*y*_*i*_ − $\widehat{y}$)		(*y*_*i*_ − $\widehat{y}$)^2^	^*b*^Standardized residuals: (*y*_*i*_ − $\widehat{y}$)/*S*_*y/x*_
1	− 0.001	− 0.00165967	0.00066		4.35E-07	0.7181
2	− 0.002	− 0.00165967	− 0.00034		1.16E-07	− 0.3704
3	− 0.001	− 0.00165967	0.00066		4.35E-07	0.7181
4	− 0.001	− 0.00165967	0.00066		4.35E-07	0.7181
5	− 0.002	− 0.00165967	− 0.00034		1.16E-07	− 0.3704
6	0.010	0.00926421	0.00074		5.41E-07	0.8009
7	0.010	0.00926421	0.00074		5.41E-07	0.8009
8	0.009	0.00926421	− 0.00026		6.98E-08	− 0.2876
9	0.009	0.00926421	− 0.00026		6.98E-08	− 0.2876
10	0.008	0.00926421	− 0.00126		1.60E-06	− 1.3761
11	0.020	0.02096838	− 0.00097		9.38E-07	− 1.0541
12	0.022	0.02096838	0.00103		1.06E-06	1.1229
13	0.020	0.02096838	− 0.00097		9.38E-07	− 1.0541
14	0.020	0.02096838	− 0.00097		9.38E-07	− 1.0541
15	0.021	0.02096838	0.00003		1.00E-09	0.0344
16	0.030	0.03189227	− 0.00189		3.58E-06	− 2.0597
17	0.032	0.03189227	0.00011		1.16E-08	0.1173
18	0.033	0.03189227	0.00111		1.23E-06	1.2058
19	0.032	0.03189227	0.00011		1.16E-08	0.1173
20	0.031	0.03189227	− 0.00089		7.96E-07	− 0.9712
21	0.043	0.04298336	0.00002		2.77E-10	0.0181
22	0.042	0.04298336	− 0.00098		9.67E-07	− 1.0704
23	0.044	0.04298336	0.00102		1.03E-06	1.1066
24	0.045	0.04298336	0.00202		4.07E-06	2.1951
25	0.044	0.04298336	0.00102		1.03E-06	1.1066
26	0.053	0.05413018	− 0.00113		1.28E-06	− 1.2302
27	0.053	0.05413018	− 0.00113		1.28E-06	− 1.2302
28	0.055	0.05413018	0.00087		7.57E-07	0.9468
29	0.055	0.05413018	0.00087		7.57E-07	0.9468
30	0.054	0.05413018	− 0.00013		1.69E-08	− 0.1417
31	0.065	0.06522127	− 0.00022		4.90E-08	− 0.2409
32	0.066	0.06522127	0.00078		6.06E-07	0.8477
33	0.066	0.06522127	0.00078		6.06E-07	0.8477
34	0.065	0.06522127	− 0.00022		4.90E-08	− 0.2409
35	0.064	0.06522127	− 0.00122		1.49E-06	− 1.3294
				SUM = 0.0000278516 degrees of freedom, *d.f.:* *p* × *n* − 2 = 7 × 5 − 2 = 33 (*S*_*y/x*_)^2^ = 0.0000278516/33 = 0.0000008440 *S*_*y/x*_ = 0.00091869 *t* (0.05, *p* − 2) = 2.571 ± *t* (0.05, *p* − 2) ×* S*_*y/x*_ = 0.0024 (dashed line)		

^a^Can be provided by statistical packages like *Excel* (it is crucial to make sure that an adequate number of significant figures is employed)

^b^*S*_*y/x*_ = Residual standard deviation or *S*_*RES*_, can be also found from Output of Excel Regression -1^st^ Table: indicated as “*Standard error*”

**Fig. 6:**
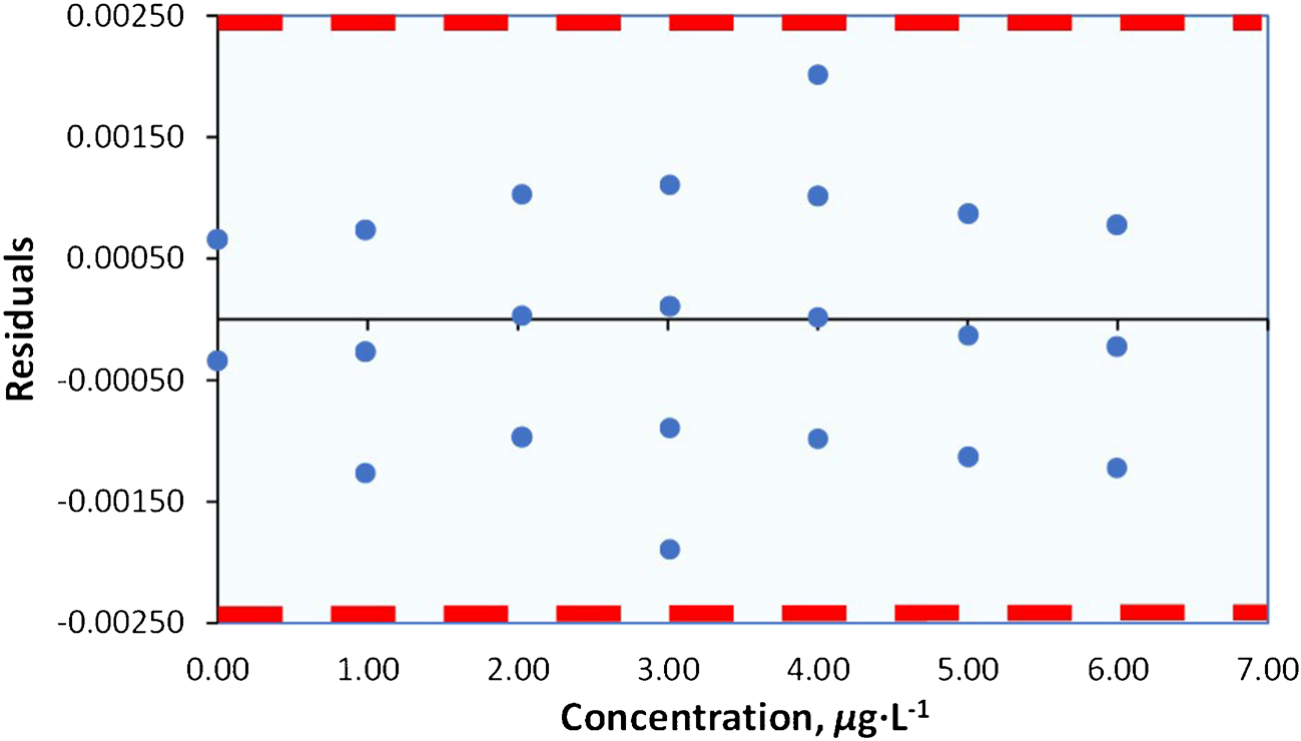
Plot of residuals *vs* Cd concentration. No evidence of outliers is observed. Dashed lines represent the ± *t* (0.05, *p*–2) ×* S*_*y/x*_.

These differences are then plotted against their respective concentration levels as shown in Fig. 6. In this plot, two horizontal dashed lines, which indicate the acceptable range of deviation for each individual data point, are drawn at ± *t* (0.05, *p*–2) × *S*_*y/x*_.

Another straightforward numerical criterion to identify potential outliers is to check if standardized residuals (a residual divided by *S*_*y/x*_ is commonly known as a standardized residual) greater than 3 are found (last column of Table 3). Similarly, there is also no indication of an outlier in this hypothetical scenario.

Note: Other more sophisticated calculation techniques include the estimation of Cook’s squared distance for each data point. However, before applying these, it is important to identify (based on the above) the suspect calibration point that could potentially be omitted.

### Procedures for linearity assessment

As mentioned before, *R*^2^ values can be misleading in the context of linearity evaluation. Examining the plot of residuals generated through linear regression of the responses against the concentrations is an option to assess the linearity. The residual plot (Fig. 6) exhibits a random pattern within the range, without any discernible systematic pattern, indicating that the linearity assumption is likely correct. However, there are instances when this visual inspection may be quite personal and open to interpretation.

Note: By plotting the ratio of the individual response values to their corresponding concentrations *vs* the concentration range could be another option (Huber’s linearity test [5]). The lower and upper limits of tolerance are established by multiplying the median value of the ratio of individual response values to their corresponding concentrations with constant factors of 0.95 and 1.05, respectively. The calibration range falls within the linear range as there are no results that exceed the tolerance limits.

To ensure the accuracy of the chosen model (Eq. 1), it is essential to employ reliable statistical tools that can confirm the assumption of linearity. This necessitates the use of robust statistical methods, such as ANalysis Of VAriance (ANOVA).

If the regression *F*-value (found in the Excel output, Table 2) is greater than the critical *F* (0.05, 1, *p* × *n*–*p*), then the regression model is considered acceptable. This indicates that the variations in the response can be explained by the model.

The ANOVA-LOF, also known as the LOF (lack of fit) test, is the most common and reliable statistical test that is applied to calibration experiments for the acceptance of the model linearity. The ratio of *F*_*LOF*_ (MS-_LackOfFit_/MS-_PureError_) is compared to critical *F* (0.05, *p*–2, *p* × *n*–*p*). If *F*_*LOF*_ is equal or less than critical *F*, it is possible to accept the hypothesis that the regression model is linear. From Table 3, we obtain the residual error sum of squares $\sum {\left({y}_{i}-\widehat{y}\right)}^{2}$ or *SS*_*RES*_ = 0.0000278516. The pure error sum of squares (*SS*_*PE*_) is equal to 0.0000244000 (see Table 4).

**Table 4:** Data treatment for the calculation of the terms *SS*_*PE*_ and *MS*_*PE*_

No	Response (*y*_*i*_)	Average response ($\overline{y }$)	*y*_*i*_ − $\overline{y }$	(*y*_*i*_ − $\overline{y }$)^2^
1	− 0.001	− 0.0014	0.0004	0.0000002
2	− 0.002		− 0.0006	0.0000004
3	− 0.001		0.0004	0.0000002
4	− 0.001		0.0004	0.0000002
5	− 0.002		− 0.0006	0.0000004
6	0.010	0.0092	0.0008	0.0000006
7	0.010		0.0008	0.0000006
8	0.009		− 0.0002	0.0000000
9	0.009		− 0.0002	0.0000000
10	0.008		− 0.0012	0.0000014
11	0.020	0.0206	− 0.0006	0.0000004
12	0.022		0.0014	0.0000020
13	0.020		− 0.0006	0.0000004
14	0.020		− 0.0006	0.0000004
15	0.021		0.0004	0.0000002
16	0.030	0.0316	− 0.0016	0.0000026
17	0.032		0.0004	0.0000002
18	0.033		0.0014	0.0000020
19	0.032		0.0004	0.0000002
20	0.031		− 0.0006	0.0000004
21	0.043	0.0436	− 0.0006	0.0000004
22	0.042		− 0.0016	0.0000026
23	0.044		0.0004	0.0000002
24	0.045		0.0014	0.0000020
25	0.044		0.0004	0.0000002
26	0.053	0.0540	− 0.0010	0.0000010
27	0.053		− 0.0010	0.0000010
28	0.055		0.0010	0.0000010
29	0.055		0.0010	0.0000010
30	0.054		0.0000	0.0000000
31	0.065	0.0652	− 0.0002	0.0000000
32	0.066		0.0008	0.0000006
33	0.066		0.0008	0.0000006
34	0.065		− 0.0002	0.0000000
35	0.064		− 0.0012	0.0000014
			SUM = 0.0000244000 = *SS*_*PE*_ *d.f.:* *p* × *n* − *p* = 7 × 5 − 7 = 28 *MS*_*PE*_ = 0.0000244000/28 = 0.000000871429	

Since the sum of squares of the residuals (*SS*_*RES*_) are divided into pure error (*SS*_*PE*_) and lack of fit (*SS*_*LOF*_), the latter term can be calculated by subtraction: *SS*_*LOF*_ = *SS*_*RES*_ − *SS*_*PE*_ = 0.0000278516 − 0.0000244000 = 0.00000345163. Mean squares (MS) for lack of fit (*MS*_*LOF*,_ *p* − 2 *d.f.*) is calculated as 0.00000345163/5 = 0.000000690325. Accordingly, the mean squares for pure error, (*MS*_*PE*_, *p* × *n* − *p d.f.*) is 0.0000244000/28 = 0.000000871429. So, *F*_*LOF*_ = *MS*_*LOF*_/*MS*_*PE*_ = 0.000000690325/0.000000871429 = 0.792 < *F*_*critical*_ (0.05 5, 28) = 2.5581.

If there is no significant lack-of-fit, it is advisable to conduct a final *t*-test to determine if the intercept significantly deviates from zero. If the calculated *t-value (*= *a*/*S*_*a*_) is equal or lower to the critical *t* (*d.f.: p* − 2, confidence limit:  99.9%, although a 95% is more common), the null hypothesis that the intercept is not significantly different from zero is accepted. From Eq. 1, we calculate *t* = 0.0017/0.0003 = 5.666 > 4.032 *t*_crit_ (0.01, 5). Therefore, the calibration curve should not be forced through zero and it is described properly by Eq. 1, as given above.

Note: Arithmetically, the decision to force zero or not can be based on the comparison of *y*-intercept (*α*) to its standard error (*S*_*a*_ = 0.000280046, Table 2). If *y*-intercept > *S*_*a*_, then *b* ≠ 0, otherwise *b* = 0.

### Calculation of a concentration, its error, and the limits of detection

Assuming that homoscedasticity is fulfilled, the regression line computed in the preceding section will be utilized for estimating the concentrations of test samples through interpolation as well as its error. Additionally, it may be employed for estimating the limit of detection of the analytical procedure. Hence, the significance lies in the random errors associated with the values of the slope (*S*_*b*_) and intercept (*S*_*a*_).

The unknown sample concentration can readily be determined by substituting the sample signal or response of 0.030 (for *k* = 5 replicates) into the regression equation (Eq. 1). This yields *x* value of 2.84024 μg⋅L^-1^. To calculate the overall error in the corresponding concentration we employ the following formula [2]:

2 $${s}_{x} =\frac{{s}_\frac{y}{x}}{b} \sqrt{\frac{1}{k}+\frac{1}{p}+\frac{{(y-\overline{y} }^{)2}}{{b}^{2}\sum_{i} {{(x}_{i}-\overline{x })}^{2}}}$$

where *k* is the number of replicate measurements we use to establish the sample’s average signal *y*, *p* is the number of calibration points, $\overline{y }$ is the average signal for the calibration standards, *x*_*i*_ and $\overline{x }$ are the individual and the mean concentrations for the calibration standards and *b* is the calculated slope from the regression equation (Eq. 1).

Confidence limits can be calculated as *μ* = *x*_*o*_ ± *t × s*_*xo*_, (*p* − 2 *d.f.*). In our case, *x*_*o*_ = 2.84024 μg⋅L^-1^, *s*_*xo*_ = 0.04833 μg⋅L^-1^, and the corresponding 95% confidence limits (*t*_*3*_ = 2.571) are 2.84024 ± 0.12425 μg⋅L^-1^. [*S*_*y/x*_ = 0.000918689, *b* = 0.01115, *k* = 5, *p* = 7, *y*(_sample signal_) = 0.030, $\overline{y }$ = 0.032, ${(y-\overline{y})}^{2}$ = 0.0000033, $\overline{x }$ = 3.004, Σ_i_(${x}_{i}-\overline{x }$)^2^ = 28.05132143].

As we have seen, the limit of detection (LOD) can be described as the concentration of the analyte that produces a signal equivalent to the blank signal, *y*_*B*_, augmented by three times the standard deviations of the blank, *s*_*B*_: LOD = *y*_*B*_ + 3*×s*_*B*_. The calculated intercept (*α*) can serve as an estimation for the value of *y*_*B*_. The unweighted least-squares method relies on the assumption that every point on the plot—including the point representing the blank or background—exhibits a variation that follows a normal distribution. This variation occurs only in the *y*-direction and its standard deviation is estimated using *S*_*y/x*_. Thus, it is suitable to substitute *S*_*y/x*_ for *s*_*B*_, when estimating the limit of detection. From previous calculations *S*_*y/x*_ = 0.000918689 and *y*_*B*_ ≈ *α* = 0.0017. Thus, LOD = 0.0017 + (3 × 0.000918689) = 0.00446 μg⋅L^−1^.

Note: It is feasible to conduct multiple repetitions of the blank experiment to acquire independent values for *s*_*B*_. These two approaches for estimating LOD should exhibit negligible differences.

For the standard additions method, the concentration of the analyte in the test sample can be calculated from the ratio of the intercept (*α*) and the slope (*b*) of the regression line (since this is extrapolated to the point on the *x*-axis at which *y* = 0). As the concentration of the sample cannot be predicted solely based on a single measured value (multiple standard additions are required) the error of the extrapolated *x*_*E*_ value is provided by:

3 $${s}_{{x}_{\rm E}} =\frac{{S}_{y/x}}{b} \sqrt{\frac{1}{n}+\frac{{\overline{y} }^{2}}{{b}^{2}\sum_{i} {{(x}_{i}-\overline{x })}^{2}}}$$

The respective confidence limits can be calculated as *x*_*E*_ ± *t* (*p* − 2) × *s*_*xE*_ (see worked example below).

The copper concentration in wastewater was determined by the method of standard additions. The following results were obtained with the aid of flame atomic absorption spectrometry: added Cu (moles L^−1^): 0, 10.0, 20.0, 30.0, 40.0, 50.0, 60.0, response (absorbance): 0.4115, 0.647, 0.8134, 1.021, 1.212 1.356, 1.667. Regression analysis provides the values of intercept *a* = 0.44, slope *b* = 0.0189. The ratio of these values (*α*/*b*) provides the concentration of 0.44/0.0189 = 23 mol⋅L^−1^. *S*_*y/x*_ = 0.024575484, $\overline{y }$ = 1.0183, $\sum_{i}{{(x}_{i}-\overline{x })}^{2}$ = 2800. Therefore, the calculated error of the extrapolated *x*_*E*_ value is: ${s}_{{x}_{\rm E}}$ = 1.408 and the confidence limits are 23 ± 2.57 × 1.408, i.e., 23.1 ± 3.6 (mol⋅L^−1^).

### Comparison of analytical methods

**Fig. 7:**
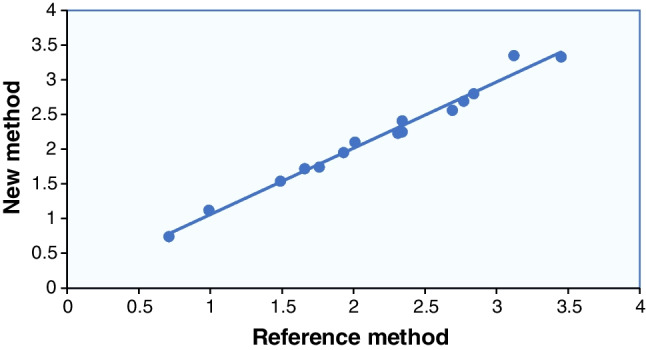
Comparison of two analytical methods with regression analysis

Typically, when comparing two methods (e.g., reference and new method, Table 5) across various levels of analyte concentrations, it is customary to follow the procedure depicted in Fig. 7.

**Table 5:** Data obtained by two analytical methods

Sample No	Reference method	New method	Mean of two methods	Difference
1	1.49	1.54	1.515	− 0.05
2	2.31	2.23	2.27	0.08
3	2.69	2.56	2.625	0.13
4	3.12	3.35	3.235	− 0.23
5	0.99	1.12	1.055	− 0.13
6	2.77	2.69	2.73	0.08
7	2.34	2.25	2.295	0.09
8	3.45	3.33	3.39	0.12
9	0.71	0.74	0.725	− 0.03
10	1.66	1.72	1.69	− 0.06
11	1.93	1.95	1.94	− 0.02
12	2.01	2.10	2.055	− 0.09
13	2.84	2.80	2.82	0.04
14	1.76	1.74	1.75	0.02
15	2.34	2.41	2.375	− 0.07
16	1.49	1.54	1.515	− 0.05
17	2.31	2.23	2.27	0.08
18	2.69	2.56	2.625	0.13
19	3.12	3.35	3.235	− 0.23
20	0.99	1.12	1.055	− 0.13
21	2.77	2.69	2.73	0.08
22	2.34	2.25	2.295	0.09
23	3.45	3.33	3.39	0.12
24	0.71	0.74	0.725	− 0.03
25	1.66	1.72	1.69	− 0.06
mean difference: *m*_*d*_: − 0.008 standard deviation of the differences: *sd*_*d*_: 0.101

If each sample produces the same outcome with both analytical protocols, the regression line depicted will have an intercept of zero, a slope of 1, and a correlation coefficient of 1. Typically, we aim to examine whether the intercept significantly deviates from zero and whether the slope significantly deviates from 1. Confidence limits for the values of constant and intercept are calculated, usually at a significance level of 95%.

Based on the calculation of the regression parameters (as in the previous worked example) the intercept is determined to be 0.103923647, with upper and lower confidence limits of − 0.066613699 and + 0.274460993, respectively, encompassing the desired value of zero. Similarly, the slope is 0.955604606244823, with a 95% confidence interval that falls within 0.880924897601987 − 1.03028431488766 including the value of 1.

Note: The focus is more on establishing the range within which the slope and intercept values are likely to fall, rather than solely relying on the correlation coefficient. The method that offers higher precision is represented on the *x*-axis of the graph while we assume that the error in the *y*-values remains constant (homoscedasticity).

**Fig. 8:**
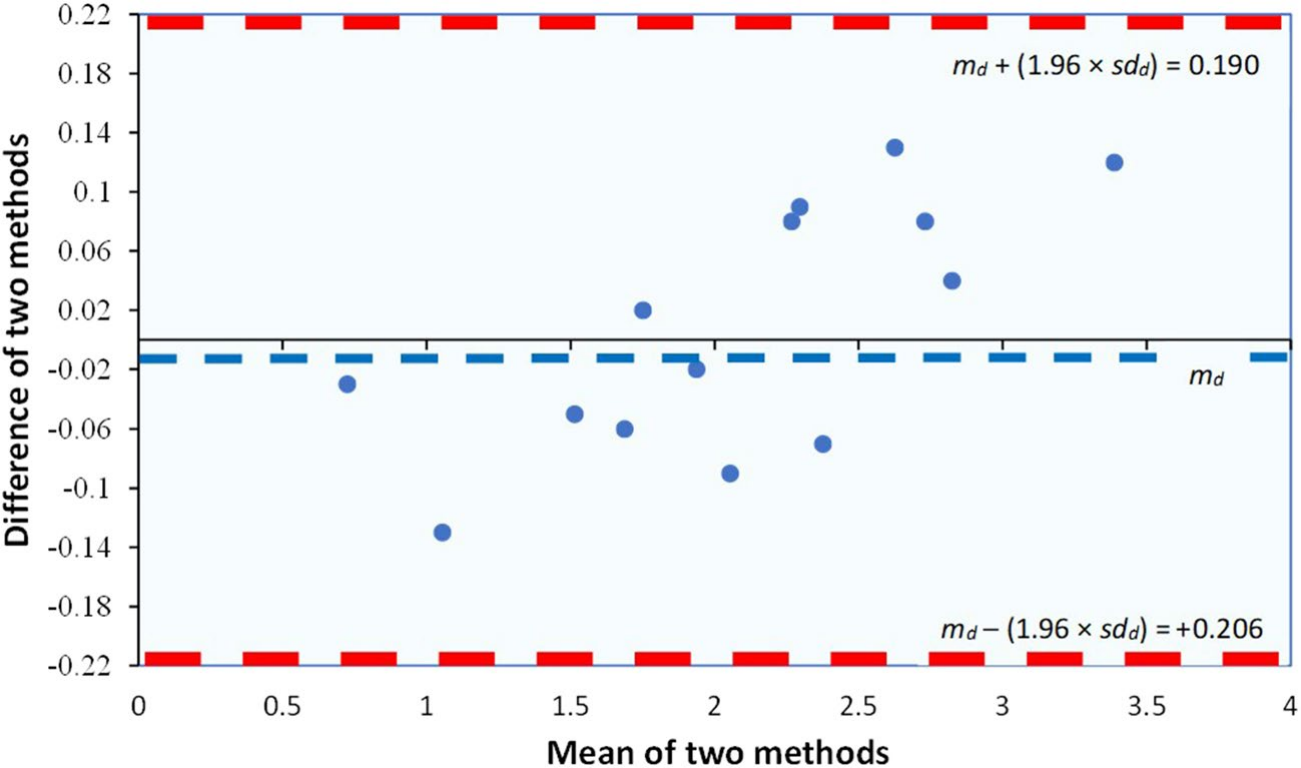
Bland and Altman plot for data from Table 5, with the representation of the limits of agreement (dotted lines), from − 1.96 *× sd*_*d*_ to + 1.96 × *sd*_*d*_. *m*_*d*_: mean difference, *sd*_*d*_: standard deviation of the differences

Bland–Altman plots serve as a valuable graphical tool, especially in clinical analysis to compare and evaluate the agreement between two sets of data obtained from different measurement techniques. Illustrated in Fig. 8, these plots visually depict the difference between two measurements on the *y*-axis and the average of those measurements on the *x*-axis. Additionally, a horizontal line is incorporated to represent the mean difference between the two measurements. Also, these plots, as already mentioned feature lines—the limits of agreement (LOA)—indicating the standard deviation, typically ± 1.96 × standard deviation of the differences (*sd*_*d*_) from the mean difference (*m*_*d*_). This enables the identification of any potential outliers within the data [24].

From the data of Table 5, *sd*_*d*_ = 0.101, so the 95% of differences will be: a) for − 1.96 × *sd*_*d*_: − 0.008 − (1.96 × 0.101) =  − 0.206, and b) for + 1.96 × *sd*_*d*_: − 0.008 + (1.96 × 0.101) = 0.190.

## Conclusions

Researchers need to realize the limits and capabilities of conventional statistics and they should bring into their chemical analysis elements of scientific judgement about the plausibility of statistics. This review focuses, mainly, on the regression and correlation to find connections between two variables, measure the connections, and to make predictions of analyte concentrations in a proper way. By way of a practical example, the *Excel* software package can easily generate a large number of statistics in a form which is digestible and easily applicable. The tutorial review benefits researchers and authors embarking on studies handling analytical measurements.
